# Impact of Freezing Delay Time on Tissue Samples for Metabolomic Studies

**DOI:** 10.3389/fonc.2016.00017

**Published:** 2016-01-28

**Authors:** Tonje H. Haukaas, Siver A. Moestue, Riyas Vettukattil, Beathe Sitter, Santosh Lamichhane, Remedios Segura, Guro F. Giskeødegård, Tone F. Bathen

**Affiliations:** ^1^Department of Circulation and Medical Imaging, Norwegian University of Science and Technology, Trondheim, Norway; ^2^Faculty of Medicine, K. G. Jebsen Center for Breast Cancer Research, Institute of Clinical Medicine, University of Oslo, Oslo, Norway; ^3^St. Olavs Hospital, Trondheim University Hospital, Trondheim, Norway; ^4^Department of Health Science, Faculty of Health and Social Science, Sør-Trøndelag University College, Trondheim, Norway; ^5^Department of Food Science, Faculty of Science and Technology, Aarhus University, Årslev, Denmark; ^6^Metabolomic and Molecular Image Laboratory, Health Research Institute INCLIVA, Valencia, Spain

**Keywords:** cancer, freezing time delay, HR MAS, metabolic profile, MR spectroscopy, metabolomics, snap-freezing, degradation

## Abstract

**Introduction:**

Metabolic profiling of intact tumor tissue by high-resolution magic angle spinning (HR MAS) MR spectroscopy (MRS) provides important biological information possibly useful for clinical diagnosis and development of novel treatment strategies. However, generation of high-quality data requires that sample handling from surgical resection until analysis is performed using systematically validated procedures. In this study, we investigated the effect of postsurgical freezing delay time on global metabolic profiles and stability of individual metabolites in intact tumor tissue.

**Materials and methods:**

Tumor tissue samples collected from two patient-derived breast cancer xenograft models (*n* = 3 for each model) were divided into pieces that were snap-frozen in liquid nitrogen at 0, 15, 30, 60, 90, and 120 min after surgical removal. In addition, one sample was analyzed immediately, representing the metabolic profile of fresh tissue exposed neither to liquid nitrogen nor to room temperature. We also evaluated the metabolic effect of prolonged spinning during the HR MAS experiments in biopsies from breast cancer patients (*n* = 14). All samples were analyzed by proton HR MAS MRS on a Bruker Avance DRX600 spectrometer, and changes in metabolic profiles were evaluated using multivariate analysis and linear mixed modeling.

**Results:**

Multivariate analysis showed that the metabolic differences between the two breast cancer models were more prominent than variation caused by freezing delay time. No significant changes in levels of individual metabolites were observed in samples frozen within 30 min of resection. After this time point, levels of choline increased, whereas ascorbate, creatine, and glutathione (GS) levels decreased. Freezing had a significant effect on several metabolites but is an essential procedure for research and biobank purposes. Furthermore, four metabolites (glucose, glycine, glycerophosphocholine, and choline) were affected by prolonged HR MAS experiment time possibly caused by physical release of metabolites caused by spinning or due to structural degradation processes.

**Conclusion:**

The MR metabolic profiles of tumor samples are reproducible and robust to variation in postsurgical freezing delay up to 30 min.

## Introduction

The field of metabolomics has the potential to fill important gaps within the knowledge of cancer biology (1). Within this field, molecular pathways and interactions are studied through the expression of small molecular compounds called metabolites. These compounds are intermediates or end products of ongoing biochemical processes, and the overall metabolic profile represents a unique fingerprint of the cellular state at a specific time point. Metabolites constitute the final level in the -omics cascade, downstream to genomics, transcriptomics, and proteomics, reflecting the combined effect of all the upstream molecular levels (2). However, the metabolic snapshot obtained from a tumor tissue specimen depends on additional factors, such as the tumor microenvironment and the polyclonality frequently observed in cancer, which introduces additional complexity for the interpretation of the metabolic information. Nevertheless, metabolic profiling of intact fresh frozen tissue is gaining popularity in clinical research, as it potentially can identify novel prognostic or predictive metabolic biomarkers or explore the abnormal biochemical activity aiming to identify novel therapeutic approaches.

Metabolomic studies using high-resolution magic angle spinning MR spectroscopy (HR MAS MRS) enables investigation of tumor tissue with minimal sample preparation, thus limiting loss of information through tissue extraction and maintaining high reproducibility (3). HR MAS MRS is also a non-destructive technique (4) shown to retain histopathological characteristics (5) and high RNA quality (6) of analyzed tissue. This technology has been used to discriminate between tumor and normal tissues in several cancers (7), but is increasingly used to explore the role of metabolomics in patient stratification for personalized oncology (8–10). In these studies, biobanks have been established after collecting tumor tissue from large patient cohorts and the association between metabolic characteristics and disease outcome has been investigated. The quality of data from such studies requires a high degree of analytical accuracy and precision, as well as highly standardized and validated protocols for sample collection, storage, and handling prior to analysis.

One of the critical points during sample collection, especially in a clinical setting, is the time period from blood supply cutoff during surgical resection until the sample is frozen for storage (freezing delay time). This interval may vary depending on the difficulty of the surgical procedure and the required tissue processing procedures, while cellular enzymatic and chemical reactions will take place and potentially cause alterations in the tissue metabolomic profile. Therefore, it is important to assess the susceptibility of these profiles to systematic variability resulting from sample handling and analysis. The main objective of this study was to investigate the metabolic effects of freezing delay time, aiming to validate the sample collection protocols normally used in biobanking for MR metabolomics studies. To minimize the impact of inter- and intratumor variability, tumor tissue was obtained from two well-characterized breast cancer xenograft models (11, 12). Furthermore, we describe the metabolic effects of snap-freezing tumor samples and the degradation pattern caused by prolonged HR MAS MRS acquisitions using human breast cancer samples. Finally, sample collection and handling procedures that ensure optimal data quality in metabolomic studies of cancer tissue are suggested.

## Materials and Methods

### Tissue Samples

#### Animal Model

The two orthotopic xenograft models MAS98.12 and MAS98.06 were established by direct transplantation of biopsy tissue from primary mammary carcinomas in immunodeficient SCID mice and thereafter passaged as previously described (11). These models have been characterized by unsupervised hierarchical clustering of intrinsic genes (13, 14) to represent basal-like (poor prognosis) and luminal-like (better prognosis) breast cancer phenotype respectively (11), and they also have distinct metabolic profiles (12, 15). Mice carrying xenograft tumors [basal-like (*n* = 3) and luminal-like (*n* = 3)] were sacrificed by cervical dislocation and tumor tissue was harvested and snap-frozen in liquid nitrogen according to the protocol below. All procedures and experiments involving animals were approved by the National Animal Research Authority and carried out according to the European Convention for the Protection of Vertebrates used for Scientific Purposes.

#### Patient Material

Breast cancer tissue samples from 14 female patients undergoing surgery at St. Olav’s Hospital (Trondheim, Norway) and Molde Hospital (Molde, Norway) were included in the study. Patients were chosen without any other prior clinical information. The biopsies were snap-frozen immediately after excision during the surgical procedure and further stored in liquid nitrogen until subsequent analyses. All patients have signed a written informed consent, and the study was approved by the Regional Ethics Committee, Central Norway.

### Experimental Design and HR MAS MRS Experiments

#### Effect of Freezing Delay Time

One tumor from each mouse was divided into pieces and left at room temperature for 0, 15, 30, 60, 90, and 120 min, prior to snap-freezing in liquid nitrogen. This procedure covers both realistic and extreme freezing time delays, which could occur in tissue harvesting procedures during breast cancer surgery. In addition, one sample was analyzed immediately after excision representing the metabolic profile of the tumor tissue without exposure to liquid nitrogen or freezing. The total number of samples analyzed for this study was 42.

Before HR MAS MRS experiments, 3 μL cold sodium formate in D_2_O (24.29 mM) was added to a leak-proof disposable 30-μL insert (Bruker, Biospin GmbH, Germany) as a shimming reference. Tissue samples were cut to fit the insert (mean sample weight 9.8 mg) on a dedicated work station designed to keep the samples frozen (16) during preparation. The insert containing the frozen sample was placed in a 4-mm diameter zirconium rotor (Bruker, Biospin GmbH, Germany) and kept at −20°C for 6–8 h before the experiments to minimize degradation.

HR MAS MRS experiments were performed on a Bruker Avance DRX600 spectrometer (Bruker, Biospin GmbH, Germany) equipped with a ^1^H/^13^C MAS probe with gradient aligned with the magic angle (Bruker, Biospin GmbH, Germany). Samples were spun at 5000 Hz and experiments run at 5°C. The samples were allowed 5 min temperature acclimatization before shimming and spectral acquisition.

Spin-echo spectra were recorded using a Carr–Purcell–Meiboom–Gill (cpmg) pulse sequence (cpmgpr1D; Bruker, L4 = 126). *T*_2_ filtering was obtained using a delay of 0.6 ms between each 180° pulse to suppress macromolecules and lipid signals and enhance signal from small molecules. This resulted in a total echo time (TE) of 77 ms. The total number of scans (NS) were 64 over a spectral width of 20 ppm (−5 to 15 ppm) with an acquisition time of 3.07 s.

#### Degradation during Prolonged HR MAS MRS Analysis

Frozen human breast cancer tissue samples were cut to fit a leak-proof 30-μL disposable insert (mean sample weight: 8.8 mg) added 3 μL of phosphate-buffered saline (PBS) based on D_2_O with trimethylsilyl propionate (TSP, 1 mM) and sodium formate (1 mM). The insert was placed in a 4-mm diameter zirconium rotor (Bruker, Biospin GmbH, Germany). Spin-echo experiments (cpmgpr1D; Bruker, L4 = 136) were run with 2 ms delay between 180° pulses, TE of 273.5 ms, spectral width of 20 ppm (−5 to 15 ppm) and NS of 256 scans (17). To evaluate the effect of prolonged HR MAS MRS experimental time, data acquisition was repeated after 1.5 h. The sample was kept spinning (5000 Hz) within the magnet at 5°C in this time interval.

### Data Preprocessing and Statistical Analysis

The FIDs were multiplied by a 0.30 Hz exponential function and Fourier transformed into 64k real points. Phase correction was performed automatically for each spectrum using TopSpin 3.1 (Bruker). Further preprocessing of the HR MAS spectra was performed in Matlab R2013b (The Mathworks, Inc., USA). Due to unavailability of a stable internal reference, human spectra were referenced to the TSP peak (0 ppm) while xenograft spectra were referenced to formate (8.46 ppm). Baseline correction was achieved by setting the minimum value of each spectrum to 0 and subtracting the lowest value. Peak alignment was performed using icoshift (18). The spectral region of interest in the human samples (2.89–4.73 ppm), which excludes the main lipid peaks, was normalized to equal total mean area, while the total spectral region (0.62–4.70 ppm) was normalized to sample weight in the xenograft spectra. In human tissue, lipid signals mainly originate from adipose tissue, and the lipid peaks may be very dominant in samples with low tumor content. Thus, the normalization accounts for differences in sample size and tumor cell content, the latter not necessary in xenograft samples with homogenous distribution of cancer cells.

To find underlying structure and main differences in the dataset, the unsupervised multivariate method principal component analysis (PCA) was used. PCA is a powerful method to decrease the complexity of collinear multivariate data, such as MR spectra, into a few principal components (PCs). PCA was performed (using PLS_Toolbox 7.5.2, Matlab, Eigenvector Research, Inc., Wenatchee, WA, USA) on xenograft spectra and human breast cancer spectra to explore the metabolic variation within samples exposed to increasing delays in postsurgical freezing and prolonged experiment time respectively.

For both cohorts, metabolite assignment was based on previous published data from HR MAS MRS analyses of breast tumors (19). Furthermore, metabolite levels were determined by integrating fixed spectral regions (performed in Matlab R2013b) corresponding to the metabolites of interest and used for univariate analysis. For metabolites with baseline strongly affected by closely resonating lipids, a linear baseline ranging from the first to the last point of the integral area was used.

Linear mixed models (LMM), an extension of linear regression, can be used to model data where several measurements from the same object are available. LMM accounts for both fixed and random effects in the modeling of the metabolite levels. Fixed effects are those that are of particular interest, e.g., effect of freezing delay time, while random effects are often not of interest but cannot be adjusted for prior to the modeling, e.g., effects originating from between subjects variation. In the current study, freezing delay time as well as type of xenograft model (basal-like or luminal-like) were set as fixed effects (continuous and categorical variable respectively), while xenograft subject was set as an random effect (without interaction term). The modeling was performed in R (20) using the “nlme” package (21).

Paired *t*-test was used to find time points were the metabolic levels had changed compared to baseline and to evaluate the effect of snap-freezing. Wilcoxon signed-rank test were performed to test the effect of prolonged experiment time on metabolite levels in human tumor tissue.

To adjust for the multiple metabolites tested, calculated *p* values were corrected for using The Benjamini Hochberg false discovery rate (FDR) in Matlab R2013b (The Mathworks, Inc., USA), and the differences were considered statistically significant for adjusted *p*-values ≤0.05.

### Histopathology and Nile Red Staining

Histopathological analysis was performed in order to evaluate the presence of viable tumor tissue and mobile lipid droplets in each individual xenograft sample. After HR MAS MRS analysis, samples were immediately frozen in liquid nitrogen. About 4 and 10 μm frozen sections were stained with hematoxylin–eosin–saffron (HES) and Nile Red as described in Ref. (22), respectively.

## Results

### Effect of Freezing Delay Time in Xenograft Tumor Tissue

**Figure 1:**
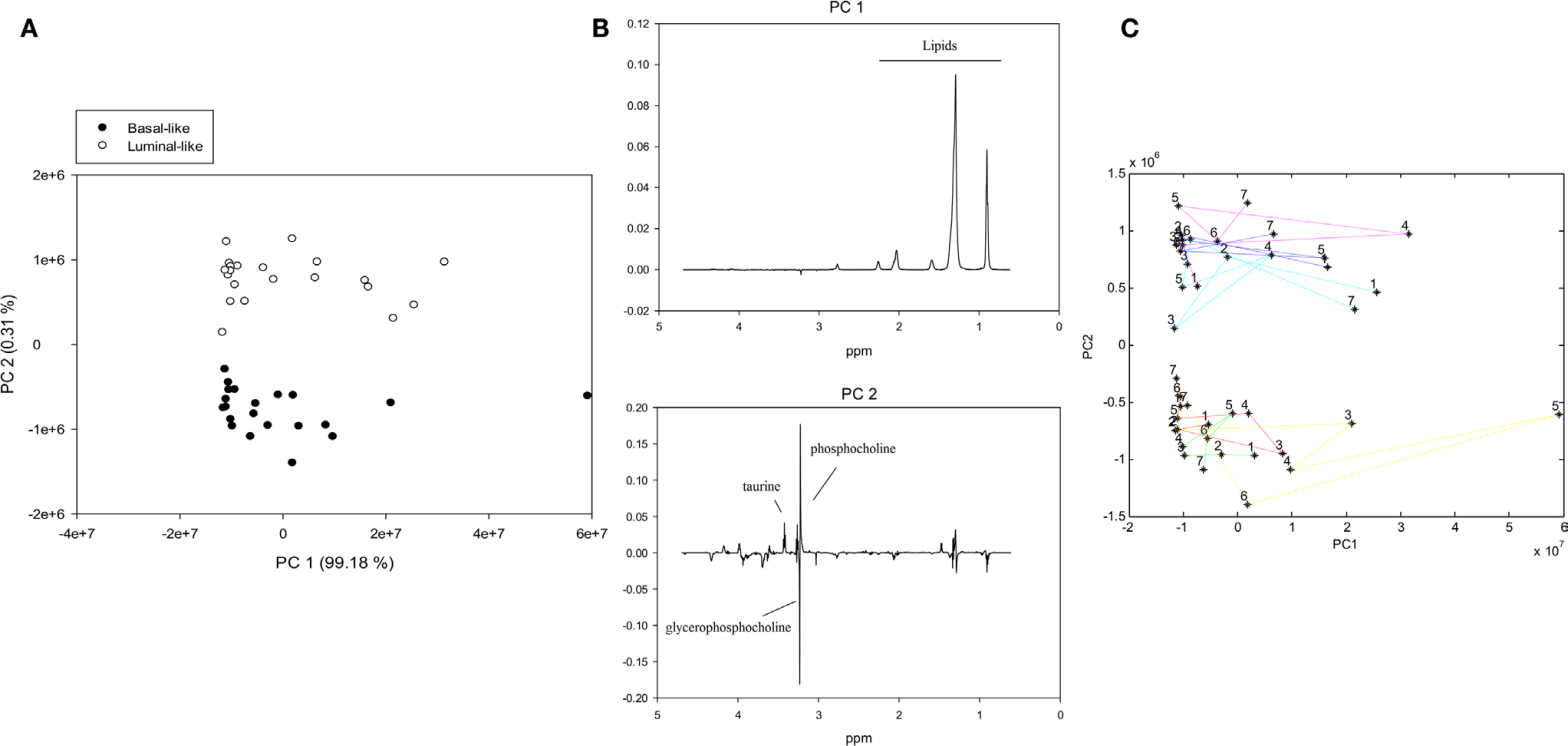
**PCA of MR spectra from xenografts tumors exposed to variable freezing delay time, (A) score plot with samples colored by xenograft type, (B) loading plots for PC1 (identifying lipid content as the most significant contributor to variability) and PC2 (identifying the metabolic difference between xenograft models as the second most significant contributor to variability), (C) PCA trajectory score plot**. Samples from the same animal are connected with colored lines and numbered according to freezing delay time: (1) not frozen, (2) 0 min, (3) 15 min, (4) 30 min, (5) 60 min, (6) 90 min, and (7) 120 min.

To examine the metabolic effect of delayed freezing, samples from the same xenograft tumor were left in room temperature for 0, 15, 30, 60, 90, and 120 min prior to freezing. A PCA score plot of the spectra from all 42 samples revealed a clear separation of basal-like and luminal-like xenograft model samples (Figure 1A). The variability between samples was predominantly attributed to the lipid content (PC1), whereas the levels of taurine, glycerophosphocholine, and phosphocholine (PC2) contributed to discrimination between the two xenograft models (Figure 1B).

A trajectory PCA score plot suggests that freezing delay time had no systematic effect on metabolic profiles (Figure 1C).

### The Impact of Freezing Delay Time on Individual Metabolites in Xenograft Samples

The LMM result for glucose was excluded due to non-normally distributed residuals. The percentage change in levels of 15 metabolites measured by HR MAS MRS in samples subject to increasing delays before freezing (*n* = 36) are shown in Table 1. After adjusting *p*-values for multiple testing, LMM revealed that three metabolites were significantly affected by type of xenograft model (basal-like and luminal-like) and four metabolites were significantly affected by delayed freezing (Table 2).

**Table 1:** **Metabolic effect of freezing delay time**.

Metabolite	ppm	15 min	30 min	60 min	90 min	120 min
Glucose	4.65	22 ± 107%	−8 ± 19%	31 ± 41%	6 ± 50%	26 ± 71%
Ascorbate	4.53	−18 ± 37%	−15 ± 20%	−25 ± 17%	−31 ± 22%	−31 ± 24%
Lactate	4.13	4 ± 44%	10 ± 24%	12 ± 29%	16 ± 27%	19 ± 45%
Tyrosine	3.99	−8 ± 32%	−10 ± 21%	−13 ± 19%	−15 ± 22%	−17 ± 29%
Glycine	3.55	−7 ± 45%	−4 ± 22%	−5 ± 25%	1 ± 45%	6 ± 62%
Myoinositol	3.53	12 ± 49%	11 ± 19%	26 ± 28%	26 ± 27%	43 ± 64%
Taurine	3.42	−7 ± 38%	−7 ± 15%	−8 ± 16%	−8 ± 20%	−4 ± 31%
Glycerophosphocholine	3.23	−9 ± 22%	−10 ± 18%	−3 ± 25%	0 ± 34%	28 ± 40%
Phosphocholine	3.22	−19 ± 24%	−7 ± 16%	1 ± 32%	7 ± 25%	34 ± 63%
Choline	3.21	6 ± 72%	20 ± 31%	56 ± 44%	62 ± 49%	111 ± 111%
Creatine	3.03	−16 ± 31%	−19 ± 18%	−28 ± 22%	−25 ± 22%	−29 ± 26%
Glutathione (GS)	2.55	−18 ± 32%	−19 ± 15%	−24 ± 25%	−35 ± 18%	−37 ± 26%
Succinate	2.41	−5 ± 35%	−13 ± 22%	−2 ± 33%	−13 ± 29%	−15 ± 38%
Glutamine	2.44	5 ± 49%	−1 ± 40%	28 ± 55%	−1 ± 22%	7 ± 54%
Glutamate	2.37	−10 ± 35%	−11 ± 17%	−20 ± 17%	−16 ± 25%	−14 ± 37%
Alanine	1.49	−7 ± 42%	9 ± 40%	2 ± 42%	17 ± 72%	23 ± 108%

*Percentage (average ± SD) increase or decrease of metabolite level in samples exposed to freezing delay time compared to samples frozen immediately after tumor collection*.

**Table 2:** **LMM-results reporting the effect of xenograft model and freezing delay time on levels of 15 metabolites**.

Metabolite	Xenograft model			Freezing time delay		

	Adj. *p*-value	Est. effect	SD	Adj. *p*-value	Est. effect	SD
Ascorbate	0.628	1.6	2.2	0.037*	−1.2	0.4
Lactate	0.849	−2.5	12.2	0.281	4.5	3.0
Tyrosine	0.059	128.3	33.4	0.343	−7.1	5.6
Glycine	0.649	−9.4	16.9	0.838	1.2	2.8
Myoinositol	0.373	−4.8	3.9	0.072	2.7	1.1
Taurine	0.025*	240.9	37.9	0.838	−2.2	7.0
Glycerophosphocholine	0.017*	−477.3	56.6	0.255	23.9	14.6
Phosphocholine	0.040*	470.3	94.3	0.255	22.7	13.8
Choline	0.068	43.5	12.5	0.002**	16.2	3.7
Creatine	0.059	−41.6	10.3	0.037*	−8.4	3.0
Glutathione (GS)	0.649	−4.1	7.3	0.005**	−6.0	1.6
Succinate	0.112	8.7	3.1	0.301	−1.0	0.7
Glutamine	0.194	12.3	6.2	0.838	0.3	0.9
Glutamate	0.322	−20.9	14.4	0.348	−3.7	3.1
Alanine	0.194	17.1	8.4	0.838	0.4	1.6

The estimated effect (Est. effect) reports each fixed factors (i.e., xenograft model or freezing time delay) influence on metabolite levels. Adjusted p-values in bold indicates that the level is significantly different from the sample frozen after 0 min (*adjusted *p* < 0.05, **adjusted *p* < 0.01).

**Figure 2:**
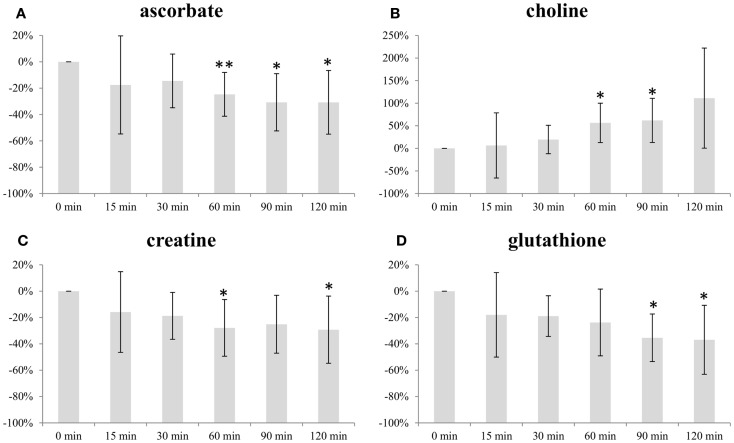
**Impact of freezing delay time on level of (A) ascorbate, (B) choline, (C) creatine, and (D) glutathione**. Metabolite integrals from samples subject to 15, 30, 60, 90, and 120 min freezing delay time compared with samples frozen immediately (0 min). * and ** indicates that the level is significantly different from the sample frozen after 0 min (*p < 0.05, **p < 0.01).

Figure 2 illustrates the change in average level of ascorbate, choline, creatine, and glutathione (GS) with increasing freezing delay time. The levels of ascorbate, creatine, and GS decreased with time. Both ascorbate and creatine levels decreased with approximately 30% within the 120 min time frame, while levels of GS were approximately 40% lower. The choline levels increased with time, reaching a level approximately 110% higher than baseline at freezing delay time of 120 min.

Ascorbate, choline, and creatine levels were significantly different from baseline sample (frozen immediately) after 60 min freezing delay time while the same was observed for GS levels after 90 min (Figure 2).

### Metabolic Effect of Freezing

**Figure 3:**
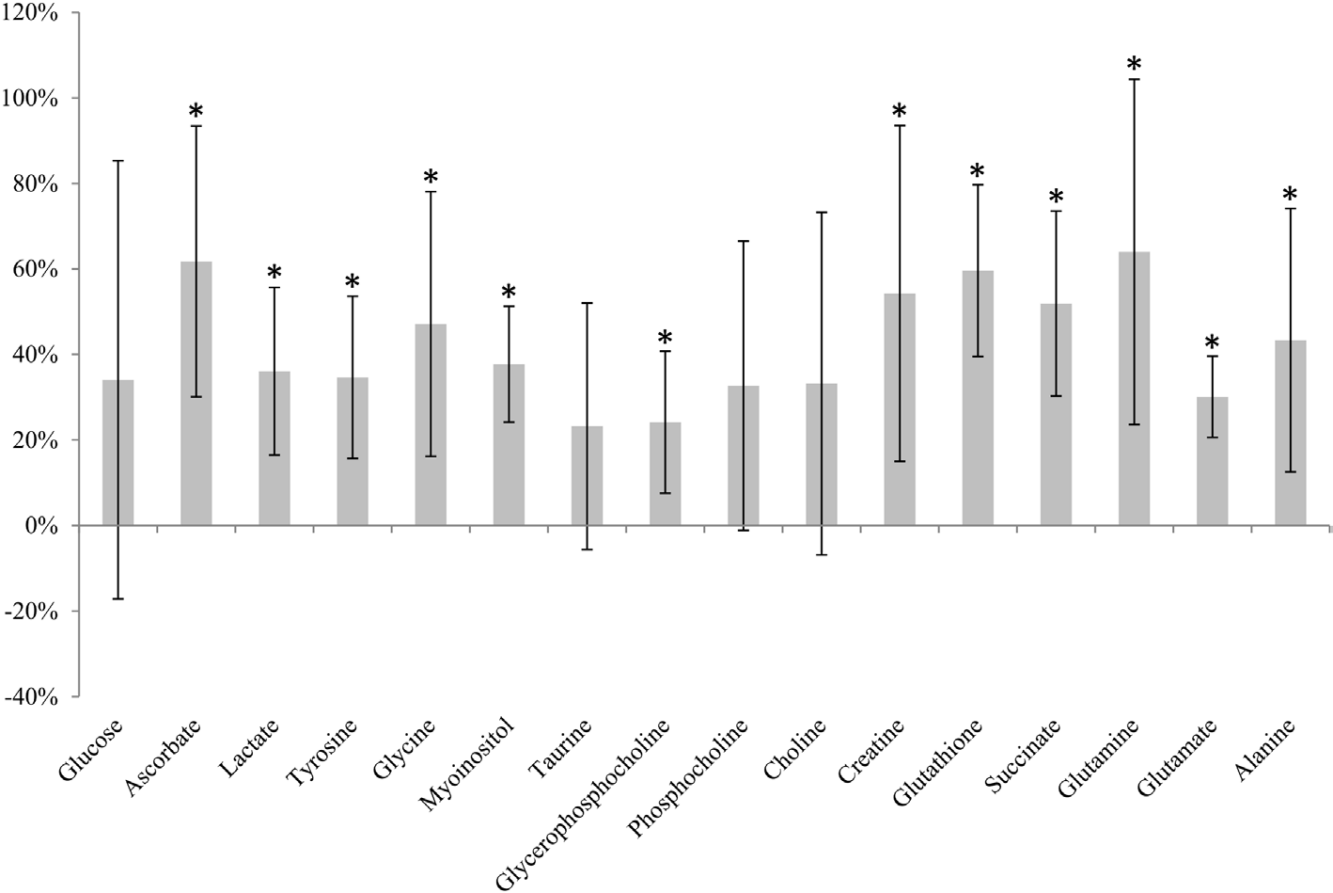
**Metabolic effect of snap-freezing**. Percentage change in metabolite levels measured in frozen samples relative to samples not frozen prior to HR MAS MRS analysis. * indicates that the level is significantly different from the sample not frozen (*adjusted *p* < 0.05).

Immediately snap-frozen samples (0 min, *n* = 6) were compared to samples analyzed directly after excision (not frozen, 0 min, *n* = 6). A clear effect of freezing compared to unfrozen tissue was seen for 12 of 16 metabolites (Figure 3). Increased levels were observed for all of these metabolites after snap-freezing.

### Histopathology

Visual inspection of HES-stained sections of xenograft samples analyzed by HR MAS MRS confirmed that the samples predominantly consisted of viable tumor tissue without significant necrosis or fibrosis. No adipose tissue or normal mammary gland tissue was observed. Due to the observed heterogeneity in lipid content of samples obtained from the same xenograft, we examined whether the lipids detected were located in adipose cells lining the tumor or in lipid droplets within the tumor. Visual inspection of the Nile Red stained histological sections showed good correlation between lipid signal intensity in spectral data and the amount of lipid detected by Nile Red staining (Figures S1 and S2 in Supplementary Material). The lipids were also observed to be located inside tumors and were therefore considered to represent mobile lipids in the cancer cells and not adipose tissue adjacent to the tumors. No systematic difference in lipid content due to delayed freezing time was observed. While Figure S1 in Supplementary Material shows a pattern of decreasing Nile Red signal with increased delay before freezing, Figure S2 in Supplementary Material shows an example where the same pattern was not observed.

### Degradation during Prolonged HR MAS Analysis

Repeated HR MAS MRS analysis of 14 human breast cancer samples was performed with 1.5 h interval to observe the metabolic effect of prolonged time in the magnet. The levels of glucose, glycine, glycerophosphocholine, and choline were found to significantly change from the first to the second acquisition (Table 3). While glucose, glycine, and choline increased, levels of glycerophosphocholine decreased with prolonged experiment time. A PCA score plot of all spectra showed that the metabolic variation between samples was higher than variation in spectra obtained from the same sample (Figure S3 in Supplementary Material).

**Table 3:** **Metabolic effect of prolonged experiment time**.

Metabolite	ppm	1.5 h	Adj. *p*-value
Glucose	4.65	21 ± 20%	0.006**
Ascorbate	4.53	−4 ± 6%	0.078
Lactate	4.15	0 ± 7%	0.903
Tyrosine	3.98	3 ± 6%	0.08
Glycine	3.56	8 ± 7%	0.006**
Myoinositol	3.54	7 ± 11%	0.08
Taurine	3.42	0 ± 5%	0.903
Glycerophosphocholine	3.23	−15 ± 12%	0.001**
Phosphocholine	3.22	−4 ± 6%	0.08
Choline	3.21	11 ± 13%	0.011*
Creatine	3.03	0 ± 6%	0.903

Percentages (average ± SD) were calculated relative to the metabolite levels (integrals) from the initial experiment. Adjusted p-values in bold indicates that the level is significantly different from the sample frozen after 0 min (*adjusted *p* < 0.05, **adjusted *p* < 0.01).

## Discussion

In this study, we evaluated the metabolic effect of freezing delay time, snap-freezing in liquid nitrogen and prolonged experimental time using HR MAS MRS. The results show that levels of HR MAS MRS visible metabolites in breast tumors are not subject to significant degradation if snap-frozen within 30 min after surgical excision.

Principal component analysis showed that differences in lipid content explained most of the variance between the samples from the two different breast cancer xenograft models. This was further examined by histopathological staining of frozen sections with Nile Red, which showed no correlation between lipid content and freezing delay time. Hence, the variability explained by lipid content most likely reflects tumor heterogeneity rather than the sample handling conditions. Furthermore, PCA clearly discriminated between samples from the two xenograft models (i.e., basal-like or luminal-like breast cancer subtype). Basal-like xenografts had higher levels of glycerophosphocholine, while luminal-like xenografts had higher levels of phosphocholine and taurine, in accordance with previously published data from these xenograft models (15). The same metabolic differences between the xenograft models were observed in LMM.

Discrimination between the two xenograft models based on overall metabolic profile did not depend on freezing delay time. Furthermore, no significant changes in individual metabolite levels were observed at 30 min past tumor excision. At 60 min, levels of three metabolites had significantly changed from baseline measurements. Thus, samples should be frozen within 30 min of resection, which in general should be sufficient when obtaining tissue biopsies during surgical procedures. Ascorbate, choline, creatine, and GS were the only metabolites exhibiting significant changes within the time frame (0–120 min) used in the current study. For the majority of metabolites, no systematic dependency on freezing time delay was observed, suggesting that intratumor heterogeneity is the predominant source of variability.

Ascorbate, also known as vitamin C, and GS are important antioxidants in animal cells that, together with other antioxidants, are responsible for eliminating reactive oxygen species (ROS) from oxidative stress (23, 24). As a consequence of high ROS levels in cancer cells, GS levels are often elevated compared to normal tissue (25). GS has also been reported to be increased in estrogen receptor (ER) negative tumors compared to ER-positive (26). ROS levels can increase as a consequence of ischemia, potentially leading to oxidative damage. It is therefore plausible that the decreased levels of GS and ascorbate reflect oxidative stressed caused by prolonged ischemia. Ascorbate levels obtained from samples frozen 60, 90, and 120 min after excision were significantly lower than the levels from samples frozen immediately. The same was observed for GS levels at 90 and 120 min of freezing delay. Consequently, biological interpretation of the levels of these antioxidants should only be considered if the experimental design of the study includes a controlled freezing delay time of <30 min.

The levels of choline increased with increasing freezing delay time. Although not significant, a similar trend was observed for the choline-containing metabolites phosphocholine and glycerophosphocholine, suggesting that ischemia affects choline metabolism. Studying the effect of hypoxia in human MDA-MB-231 breast cancer cell and tumors, Jiang et al. detected higher concentrations of total choline-containing metabolites (tCho; composed of phosphocholine, glycerophosphocholine, and free choline), mainly contributed by phosphocholine, in hypoxic regions (27). Altered choline metabolism is considered an emerging hallmark in malignant transformation (28). A major component of mammalian cell membranes, phosphatidylcholine (PtdCho), is synthesized from choline, thus making choline and choline-containing intermediates essential for the increased proliferation observed in tumor cells. Several *ex vivo* breast cancer studies using HR MAS MRS have detected increased concentrations of choline, phosphocholine, and glycerophosphocholine in tumor tissue compared to non-involved breast tissue (19, 29, 30). Differences in tCho have been found to have predictive value for the 5-year survival of breast cancer patients receiving neoadjuvant chemotherapy (31) and higher choline concentrations have been found in core needle biopsies from patients that are ER- and/or PgR-negative compared to ER- and/or PgR-positive patients (10). Delays in freezing time up to 30 min had no significant impact on choline levels. While choline levels at 60 and 90 min delay were significantly increased, this was not observed at 120 min (*p* = 0.065), probably due to variability within these last measurements. However, because of the biological relevance of choline metabolism in cancer, this trend of increasing levels with freezing delay time emphasize the importance of reporting and controlling sample handling to limit possible effects.

Levels of creatine significantly decreased as a result of prolonged time before freezing, where 60 min was found to be the first time point significantly different from samples frozen directly after exiting. Creatine is involved in energy storage through formation of phosphocreatine and thus functions as a carrier of energy within cells. Decreasing levels of creatine (or phosphocreatine) could be suggestive of energy depletion caused by ischemia. Several studies use creatine for calculation of metabolic ratios to allow for comparable quantities between samples (10, 32–34) and in studies of breast cancer tissue, higher level of this metabolite have been correlated to ER-positive (35) and PgR-positive tumors (15). As the tendency of decreasing levels is seen from the initial time point, it is important to keep the time before freezing minimal to allow the usage of ratios involving creatine.

Rapid metabolic phenotyping in operating theaters of unfrozen tissue has been proposed to facilitate real-time diagnostics and further aid decision making during surgery (36). To evaluate the metabolic effect of snap-freezing, tumor tissue was analyzed by HR MAS MRS without any exposure to liquid nitrogen and compared to tissue from the same xenografts that were immediately frozen after excision. Freezing was found to significantly increase the level of 12 metabolites. In previous studies, the freezing of rat kidney and liver tissue has reportedly led to increased amounts of amino acids (37, 38) and decreased contents of choline, glycerophosphocholine, glucose, myoinositol, trimethylamine N-oxide (TMAO), and taurine (38) using HR MAS MRS. The increased levels of multiple metabolites observed in the current study might be caused by intracellular lysis releasing metabolites. Metabolites bound to cellular molecules or compartments are more restricted and thus less MR-visible. If these metabolites are released as a consequence of freezing, HR MAS MRS will detect higher levels than in unfrozen tissue as found here. The findings underpin that studies of fresh and frozen tissue are not directly comparable. Although the effect of freezing was significant for the majority of metabolites, we believe that analyzing fresh tissue samples is neither feasible nor optimal in the current clinical and research setting. Care must therefore be taken not to compare metabolic information obtained in unfrozen samples with data from frozen biobank tissue.

We also examined the effect on the metabolic profile of prolonged HR MAS MRS analysis. After the first acquisition, the sample was kept spinning inside the magnet and reanalyzed after 1.5 h. The level of four metabolites was found to differ significantly from the initial acquisition. Glucose, glycine, and choline were found to increase with time, while glycerophosphocholine decreased. Similar effects on glycine, choline, and glycerophosphocholine levels have been observed in lung cancer tissue (39) and in brain tumor tissue (40) supporting the current findings. As Rocha et al. describe, the changes might be caused by spinning effects causing release of bound metabolites or due to ongoing metabolic activity (39). Importantly, these metabolic effects should be considered for quantitative two-dimensional HR MAS MRS studies where long acquisition time is required.

In conclusion, this study confirms that HR MAS MRS metabolic profiles are robust to metabolic changes due to delayed freezing within a timeframe of 30 min. This allows biological interpretation of metabolic profiles, including metabolites involved in protection against ROS formation/oxidative stress, such as GS and ascorbate, as well as evaluation of the levels of creatine and choline-containing metabolites. Within the 30 min freezing delay time window, the effect of structural or biochemical degradation on metabolic profiles is insignificant. A clear effect of freezing was observed for most of the detected metabolites. However, this step in sample handling is considered essential for biobanking and research purposes. The study also identified moderate metabolic consequences of prolonged HR MAS experiment time, and thus, the protocol should be designed to keep experiment time to a minimum.

## Author Contributions

Author contributions included: study design (TH, SM, BS, and TB), data acquisition (SM, TH, and SL), data analysis (TH, SM, RV, SL, and RS), data interpretation (TH, SM, GG, and TB), and drafting the manuscript (TH, SM, and TB). All authors contributed in revising the manuscript.

## Conflict of Interest Statement

The authors declare that the research was conducted in the absence of any commercial or financial relationships that could be construed as a potential conflict of interest.

## References

[1] Patel S Ahmed S. Emerging field of metabolomics: big promise for cancer biomarker identification and drug discovery. J Pharm Biomed Anal (2015) 107:63–74. 10.1016/j.jpba.2014.12.020 25569286

[2] Zhang A-H Sun H Qiu S Wang X. Metabolomics in noninvasive breast cancer. Clin Chim Acta (2013) 424:3–7. 10.1016/j.cca.2013.05.003 23669185

[3] Pan Z Raftery D Comparing and combining NMR spectroscopy and mass spectrometry in metabolomics. Anal Bioanal Chem (2007) 387(2):525–7. 10.1007/s00216-006-0687-8 16955259

[4] Bathen TF Sitter B Sjøbakk TE Tessem M-B Gribbestad IS. Magnetic resonance metabolomics of intact tissue: a biotechnological tool in cancer diagnostics and treatment evaluation. Cancer Res (2010) 70(17):6692–6. 10.1158/0008-5472.CAN-10-0437 20699363

[5] Bathen T Geurts B Sitter B Fjøsne H Lundgren S Buydens L Feasibility of MR metabolomics for immediate analysis of resection margins during breast cancer surgery. PLoS One (2013) 8(4):e61578. 10.1371/journal.pone.0061578 23613877 PMC3629170

[6] Bertilsson H Tessem M-B Flatberg A Viset T Gribbestad I Angelsen A Changes in gene transcription underlying the aberrant citrate and choline metabolism in human prostate cancer samples. Clin Cancer Res (2012) 18(12):3261–9. 10.1158/1078-0432.CCR-11-2929 22510345

[7] Moestue S Sitter B Bathen T Tessem M-B Gribbestad I. HR MAS MR spectroscopy in metabolic characterization of cancer. Curr Top Med Chem (2011) 11(1):2–26. 10.2174/156802611793611869 20809888

[8] Giskeødegard GF Bertilsson H Selnæs KM Wright AJ Bathen TF Viset T Spermine and citrate as metabolic biomarkers for assessing prostate cancer aggressiveness. PLoS One (2013) 8(4):e62375. 10.1371/journal.pone.0062375 23626811 PMC3633894

[9] Giskeødegård G Lundgren S Sitter B Fjøsne HE Postma G Buydens L Lactate and glycine – potential MR biomarkers of prognosis in estrogen receptor-positive breast cancers. NMR Biomed (2012) 25(11):1271–9. 10.1002/nbm.2798 22407957

[10] Choi J Baek H-M Kim S Kim M Youk J Moon H HR-MAS MR spectroscopy of breast cancer tissue obtained with core needle biopsy: correlation with prognostic factors. PLoS One (2012) 7(12):e51712. 10.1371/journal.pone.0051712 23272149 PMC3522710

[11] Bergamaschi A Hjortland G Triulzi T Sørlie T Johnsen H Ree A Molecular profiling and characterization of luminal-like and basal-like *in vivo* breast cancer xenograft models. Mol Oncol (2009) 3(5):469–82. 10.1016/j.molonc.2009.07.003 19713161 PMC5527532

[12] Grinde MT Moestue SA Borgan E Risa Ø Engebraaten O Gribbestad IS. 13C High-resolution-magic angle spinning MRS reveals differences in glucose metabolism between two breast cancer xenograft models with different gene expression patterns. NMR Biomed (2011) 24(10):1243–52. 10.1002/nbm.1683 21462378

[13] Sørlie T Perou C Tibshirani R Aas T Geisler S Johnsen H Gene expression patterns of breast carcinomas distinguish tumor subclasses with clinical implications. Proc Natl Acad Sci U S A (2001) 98(19):10869–74. 10.1073/pnas.191367098 11553815 PMC58566

[14] Sørlie T Tibshirani R Parker J Hastie T Marron J Nobel A Repeated observation of breast tumor subtypes in independent gene expression data sets. Proc Natl Acad Sci U S A (2003) 100(14):8418–23. 10.1073/pnas.0932692100 12829800 PMC166244

[15] Moestue S Borgan E Huuse E Lindholm E Sitter B Børresen-Dale A-L Distinct choline metabolic profiles are associated with differences in gene expression for basal-like and luminal-like breast cancer xenograft models. BMC Cancer (2010) 10(1):433. 10.1186/1471-2407-10-433 20716336 PMC2931488

[16] Giskeødegård G Cao M Bathen T High-resolution magic-angle-spinning NMR spectroscopy of intact tissue. In: Bjerrum Jacob T editor. Metabonomics: Methods and Protocols. New York: Springer (2015). p. 37–50. 10.1007/978-1-4939-2377-9_4 25677145

[17] Cao M Lamichhane S Lundgren S Bofin A Fjøsne H Giskeødegård G Metabolic characterization of triple negative breast cancer. BMC Cancer (2014) 14(1):941. 10.1186/1471-2407-14-941 25495193 PMC4295321

[18] Savorani F Tomasi G Engelsen S. icoshift: a versatile tool for the rapid alignment of 1D NMR spectra. J Magn Reson (2010) 202(2):190–202. 10.1016/j.jmr.2009.11.012 20004603

[19] Sitter B Sonnewald U Spraul M Fjösne H Gribbestad I. High-resolution magic angle spinning MRS of breast cancer tissue. NMR Biomed (2002) 15(5):327–37. 10.1002/nbm.775 12203224

[20] R Core Team. R: A Language and Environment for Statistical Computing. Vienna: R Foundation for Statistical Computing (2012).

[21] Pinheiro J Bates D DebRoy S Sarkar D R Core Team nlme: Linear and Nonlinear Mixed Effects Models. R Package Version 3.1-117 (2014). Available from: http://CRAN.R-project.org/package=nlme

[22] Opstad KS Bell BA Griffiths JR Howe FA. An investigation of human brain tumour lipids by high-resolution magic angle spinning 1H MRS and histological analysis. NMR Biomed (2008) 21(7):677–85. 10.1002/nbm.1239 18186027

[23] Gorrini C Harris IS Mak TW. Modulation of oxidative stress as an anticancer strategy. Nat Rev Drug Discov (2013) 12(12):931–47. 10.1038/nrd4002 24287781

[24] Bánhegyi G Braun L Csala M Puskás F Mandl J. Ascorbate metabolism and its regulation in animals. Free Radic Biol Med (1997) 23(5):793–803. 10.1016/S0891-5849(97)00062-2 9296457

[25] Gamcsik MP Kasibhatla MS Teeter SD Colvin OM. Glutathione levels in human tumors. Biomarkers (2012) 17(8):671–91. 10.3109/1354750X.2012.715672 22900535 PMC3608468

[26] Tang X Lin C-C Spasojevic I Iversen ES Chi J-T Marks JR. A joint analysis of metabolomics and genetics of breast cancer. Breast Cancer Res (2014) 16(4):415. 10.1186/s13058-014-0415-9 25091696 PMC4187326

[27] Jiang L Greenwood TR Artemov D Raman V Winnard PT Heeren RM Localized hypoxia results in spatially heterogeneous metabolic signatures in breast tumor models. Neoplasia (2012) 14(8):732–41. 10.1593/neo.12858 22952426 PMC3431180

[28] Glunde K Bhujwalla Z Ronen S. Choline metabolism in malignant transformation. Nat Rev Cancer (2011) 11(12):835–48. 10.1038/nrc3162 22089420 PMC4337883

[29] Sitter B Lundgren S Bathen T Halgunset J Fjosne H Gribbestad I. Comparison of HR MAS MR spectroscopic profiles of breast cancer tissue with clinical parameters. NMR Biomed (2006) 19(1):30–40. 10.1002/nbm.992 16229059

[30] Gribbestad IS Sitter B Lundgren S Krane J Axelson D Metabolite composition in breast tumors examined by proton nuclear magnetic resonance spectroscopy. Anticancer Res (1998) 19(3A):1737–46. 10470108

[31] Cao M Sitter B Bathen T Bofin A Lønning P Lundgren S Predicting long-term survival and treatment response in breast cancer patients receiving neoadjuvant chemotherapy by MR metabolic profiling. NMR Biomed (2012) 25(2):369–78. 10.1002/nbm.1762 21823183

[32] Choi JS Baek H-M Kim S Kim MJ Youk JH Moon HJ Magnetic resonance metabolic profiling of breast cancer tissue obtained with core needle biopsy for predicting pathologic response to neoadjuvant chemotherapy. PLoS One (2013) 8(12):e83866. 10.1371/journal.pone.0083866 24367616 PMC3868575

[33] van Asten JJ Vettukattil R Buckle T Rottenberg S van Leeuwen F Bathen TF Increased levels of choline metabolites are an early marker of docetaxel treatment response in BRCA1-mutated mouse mammary tumors: an assessment by ex vivo proton magnetic resonance spectroscopy. J Transl Med (2015) 13(1):114. 10.1186/s12967-015-0458-4 25890200 PMC4404119

[34] Vettukattil R. Preprocessing of raw metabonomic data. Methods Mol Biol (2015) 1277:123–36. 10.1007/978-1-4939-2377-9_10 25677151

[35] Giskeødegård G Grinde M Sitter B Axelson D Lundgren S Fjøsne H Multivariate modeling and prediction of breast cancer prognostic factors using MR metabolomics. J Proteome Res (2010) 9(2):972–9. 10.1021/pr9008783 19994911

[36] Kinross JM Holmes E Darzi AW Nicholson JK Metabolic phenotyping for monitoring surgical patients. Lancet (2011) 377(9780):1817–9. 10.1016/S0140-6736(11)60171-2 21596428

[37] Middleton DA Bradley DP Connor SC Mullins PG Reid DG. The effect of sample freezing on proton magic-angle spinning NMR spectra of biological tissue. Magn Reson Med (1998) 40(1):166–9. 10.1002/mrm.1910400122 9660567

[38] Waters N Garrod S Farrant R Haselden J Connor S Connelly J High-resolution magic angle spinning 1 H NMR spectroscopy of intact liver and kidney: optimization of sample preparation procedures and biochemical stability of tissue during spectral acquisition. Anal Biochem (2000) 282(1):16–23. 10.1006/abio.2000.4574 10860494

[39] Rocha CM Barros AS Gil AM Goodfellow BJ Humpfer E Spraul M Metabolic profiling of human lung cancer tissue by 1H high resolution magic angle spinning (HRMAS) NMR spectroscopy. J Proteome Res (2009) 9(1):319–32. 10.1021/pr9006574 19908917

[40] Opstad KS Bell BA Griffiths JR Howe FA An assessment of the effects of sample ischaemia and spinning time on the metabolic profile of brain tumour biopsy specimens as determined by high-resolution magic angle spinning 1H NMR. NMR Biomed (2008) 21(10):1138–47. 10.1002/nbm.1296 18666093

