# Supplementary figures and images for: Impact of Freezing Delay Time on Tissue Samples for Metabolomic Studies
Source: Front Oncol. 2016 Jan 28;6:17. doi: 10.3389/fonc.2016.00017 (PMC4730796; doi:10.3389/fonc.2016.00017)

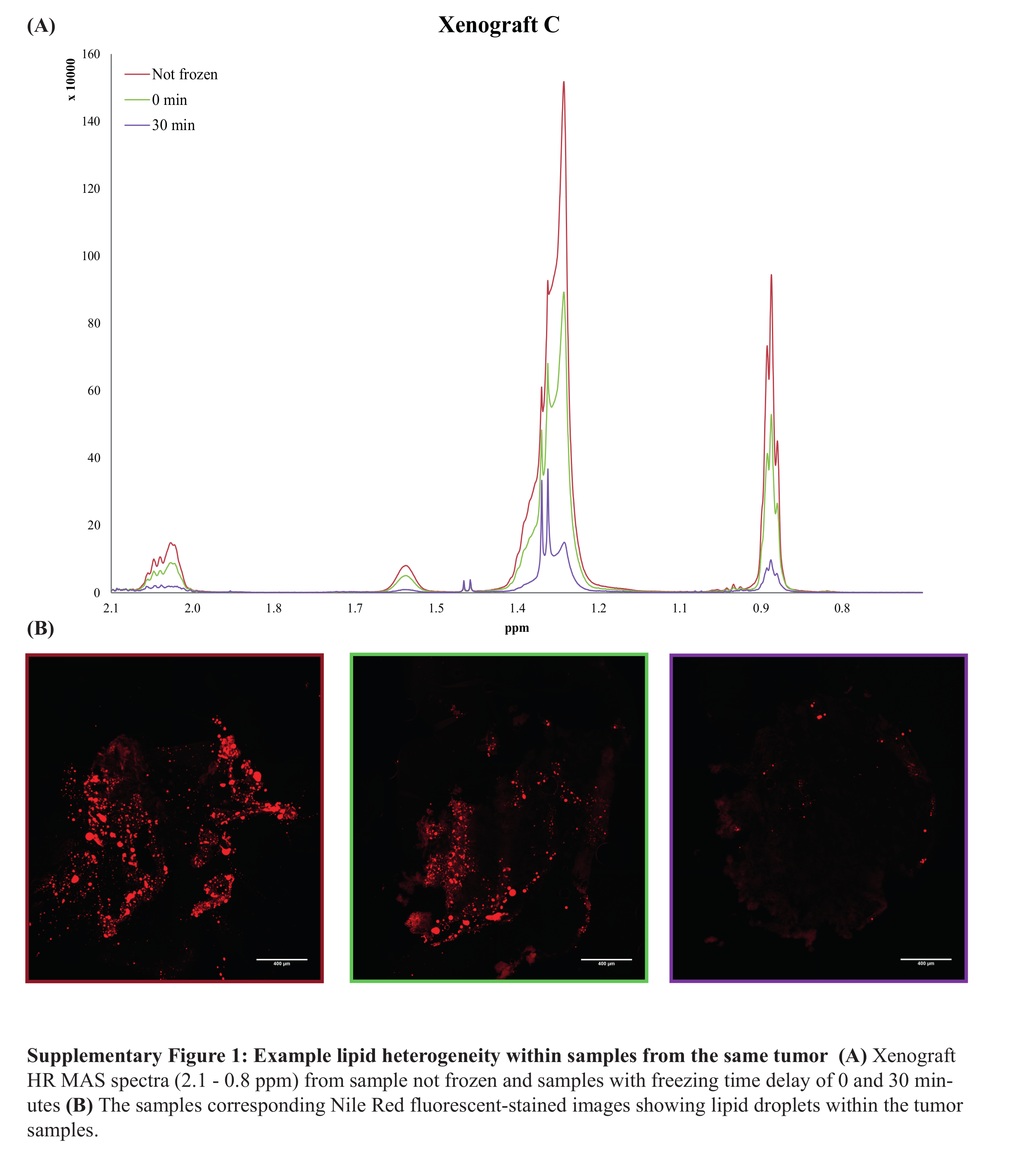

Supplement: Supplementary file 1 [file Image_1.TIF]

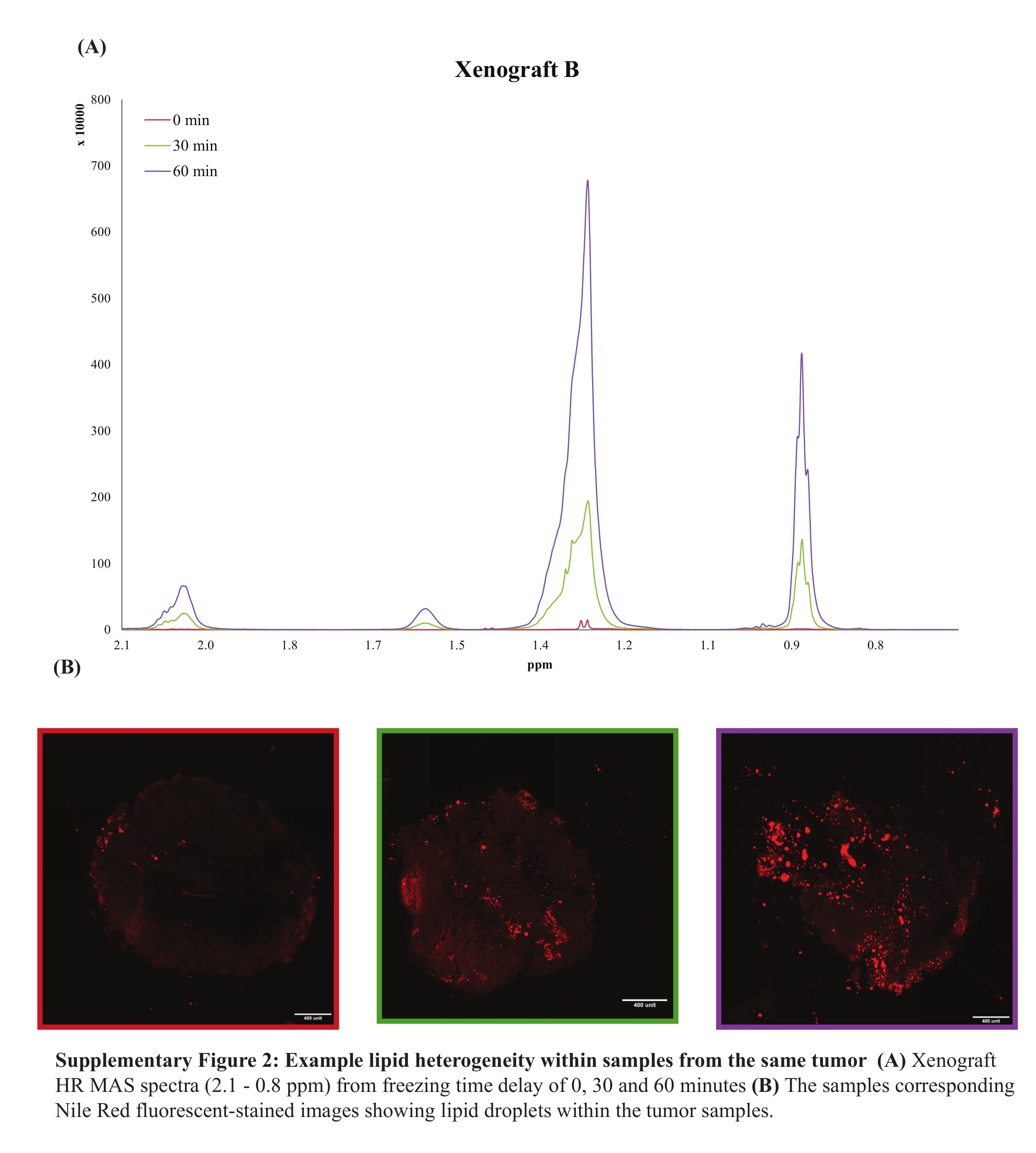

Supplement: Supplementary file 2 [file Image_2.TIF]

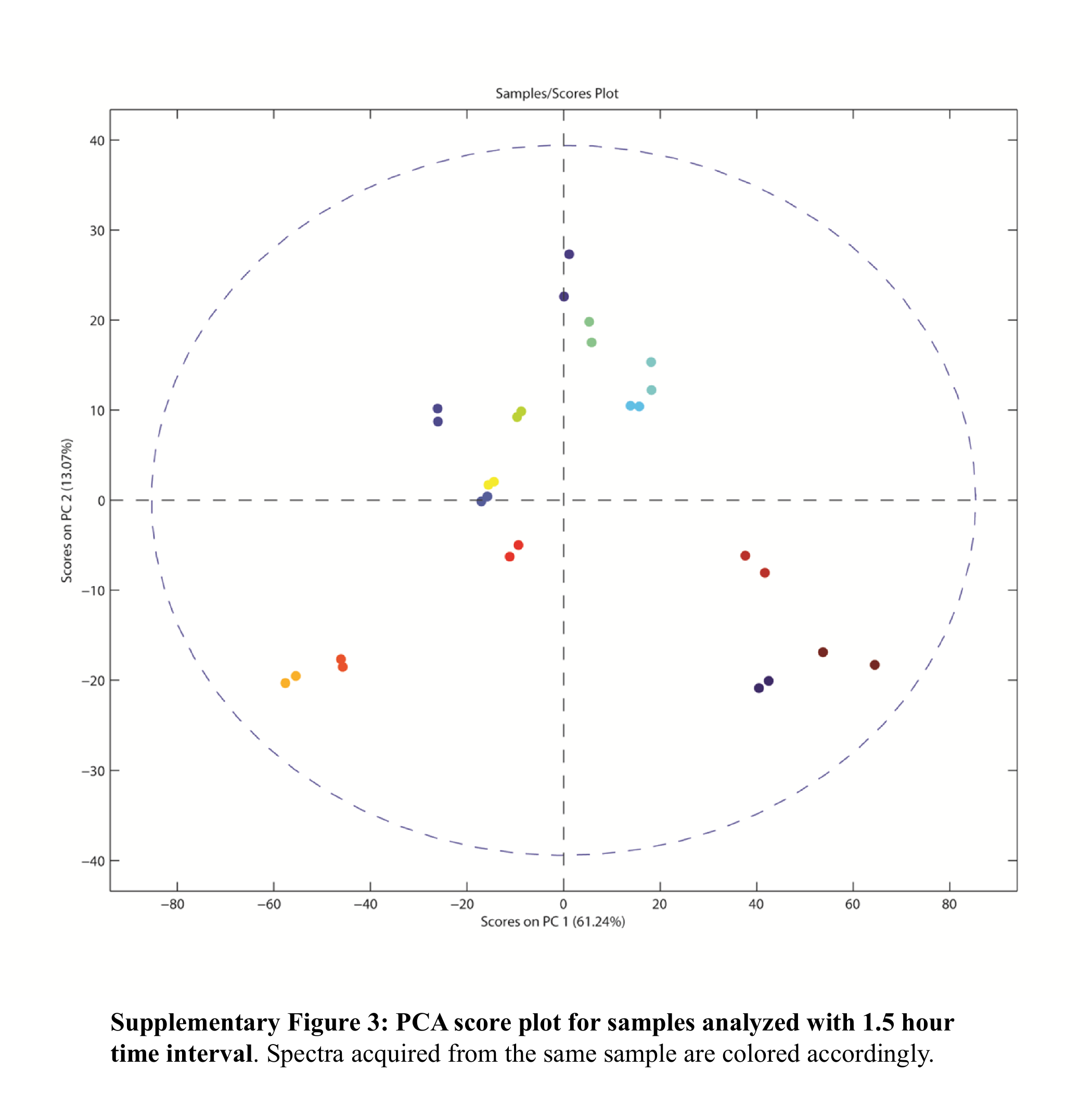

Supplement: Supplementary file 3 [file Image_3.TIF]
